# Novel Grey Body for Accurate Radiometric Measurements

**DOI:** 10.3390/mi14050974

**Published:** 2023-04-29

**Authors:** Moshe Avraham, Gady Golan, Yael Nemirovsky

**Affiliations:** 1 Electrical and Computer Engineering Department, Technion—Israel Institute of Technology, Haifa 32000, Israel; 2 Department of Electrical Engineering and Electronics, Ariel University, Ariel 40700, Israel

**Keywords:** IR sensor, radiometer, blackbody, grey body, emissivity, perforated screen, TMOS

## Abstract

This study presents an original approach on how to generate a radiator with an emissivity less than one by using a conventional blackbody and a screen with a defined area density of holes. This is needed for the calibration of infrared (IR) radiometry, which is a very useful form of temperature measurement in industrial, scientific, and medical applications. One of the major sources of errors in IR radiometry is the emissivity of the surface being measured. Emissivity is a physically well-defined parameter, but in real experiments, it may be influenced by many factors: surface texture, spectral properties, oxidation, and aging of surfaces. While commercial blackbodies are prevalent, the much-needed grey bodies with a known emissivity are unavailable. This work describes a methodology for how to calibrate radiometers in the lab or in the factory or FAB using the “screen approach” and a novel thermal sensor dubbed Digital TMOS. The fundamental physics required to appreciate the reported methodology is reviewed. The linearity in emissivity of the Digital TMOS is demonstrated. The study describes in detail how to obtain the perforated screen as well as how to do the calibration.

## 1. Introduction

This work proposes how to calibrate radiometers, blackbodies, and grey bodies (also known as grey-bodies). The calibration is based on a series of perforated screens and a commercial blackbody as well as a high-performance IR thermal sensor dubbed Digital TMOS (thermal-metal-oxide-semiconductor). We start with a brief review of the basic physics, defining Plank’s radiation law and the ideal blackbody and grey body [1,2]. We remind the reader about the concept of emissivity and why it introduces a major error during calibration [3,4,5].

Planck’s radiation law describes the spectral radiance by a perfect body at a temperature T for a given wavelength:

(1) $$W_{\lambda }\left[\frac{Watt}{\mu m\cdot \mathrm{sr}\cdot m^{2}}\right]=\frac{2hc^{2}}{\lambda^{5}}\frac{1}{\exp\left(\frac{hc}{\lambda kT}\right)-1}$$

where *c* is the speed of light 2.988 × 10^8^ [m/s], *h* is the Planck’s constant that is equal to 6.625 × 10^−34^ [J/s], *k* is Boltzmann’s constant that is equal to 1.38 × 10^−23^ [J/K], *λ* is the wavelength in microns, and T is the temperature in Kelvin of the surface area in m^2^.

For an integrated optical bandpass filter, the expression for the total exitance is:

(2) $$W_{\lambda 1-\lambda 2}\left[\frac{Watt}{m^{2}}\right]=\overset{\lambda 2}{\underset{\lambda 1}{\int }}\frac{2\pi hc^{2}}{\lambda^{5}}\frac{1}{\exp\left(\frac{hc}{\lambda kT}\right)-1}d\lambda$$

where λ1-λ2 is the wavelength bandpass of the optical filter.

A body that follows Planck’s radiation law is termed a perfect blackbody. IR radiometers measure the amount of power emitted by an object in an infrared band. They calculate temperature based on this measured power and the material’s emissivity.

Emissivity is a factor between 0 and 1 which multiplies Plank’s radiation law. It is the ratio of the radiation emitted from the surface of a body to the theoretical emission of an ideal blackbody of the same size and shape according to Plank Law. 

Every material radiates energy. The amount of radiated power is dependent on the material’s temperature and the material’s emissivity. An ideal blackbody has an emissivity = 1. A grey body has constant emissivity within the measurement IR band pass filter. 

There are two types of blackbodies: cavity and extended area. For calibration, an extended area blackbody is preferred since the measured signal from cavity type radiometers depends on the geometry of the cavity as well as the observation angle. Extended area blackbodies are made of a heated, high-conductivity metallic plate, which is covered by a black paint. A calibrated resistance temperature detector (RTD) made of platinum controls the temperature of the metallic plate.

Blackbody manufacturers strive to provide an ideal blackbody with an emissivity as close to 1 as possible, which is theoretically not achievable because such an ideal blackbody does not emit. In fact, state-of-the-art extended area blackbody manufacturers provide blackbodies with an emissivity of 0.98 ± 0.02 [6]. 

Emissivity is the primary source of error in IR temperature measurement since what is not emitted is reflected [7]. To account for the effect of reflected energy, Kirchhoff’s law is applied: the emitted, reflected, and transmitted energy are equal to 1.

(3) α+r+τ=1

where *α* is a measure of how much of the radiation is absorbed by the body, reflectivity *r* is a measure of how much is reflected, and transmissivity *τ* is a measure of how much passes through. A perfect mirror has a reflectivity of unity and an emissivity of zero. A perfect blackbody has an emissivity of unity and a reflectivity of zero. The reflected energy depends on the ambient temperature as well as on sources of IR radiation “seen” by the blackbody.

There are no blackbody manufacturers that provide a “grey body” with known lower surface emissivity. This raises the question of how to test, calibrate, and measure radiometer device performance with a target that has emissivity lower than 1 or not approximately 1. For example, industrial composite materials such as carbon-fiber have a typical emissivity of 0.85 [8].

Industrial factories monitor the manufacturing process with remote temperature sensing using black tape on the monitored surface. The black tape is calibrated, and its emissivity is measured to be ~0.95. However, tapes with well-calibrated emissivity in a wide range of values are not available. Military equipment suppliers provide low-emissivity tape, lower than 0.3, to camouflage military systems and equipment in the dark [9]. Such tapes are not available for everyone.

Testing the calibrated sensor only with a blackbody with an emissivity close to 1 can yield large inaccuracy in temperature measurement in cases where measuring surfaces with an emissivity lower than 1, if the emissivity compensation is not good enough. Therefore, using only a blackbody for testing infrared is not recommended for use in cases with different surface emissivity values. 

One suggestion to calibrate or measure grey bodies with high temperature accuracy is to put on the blackbody surface a tape with known emissivity and low thermal capacity. This approach suffers from two major disadvantages. The glue of the tape may cause damage to the blackbody surface and the surface paint by reducing the surface emissivity. Moreover, the emissivity is limited to the available tapes on the market, so only limited emissivity values are possible. 

In this work, we suggest a simple and convenient way to implement grey bodies with known effective emissivity values. We need a perforated screen with well-defined holes, a high-quality commercial blackbody, and high-performance IR thermal sensors, dubbed Digital TMOS, which is highly linear in emissivity. The requirements of these components are described in Section 2, Section 3 and Section 4. Appendix A is based on the basic physics concepts discussed above. Other methods to measure emissivity are discussed in Appendix B.

## 2. The “Screen Approach”-Measurement Setup and Methodology

With a perforated screen, the effective emissivity of the surface is simply the hole density area of the surface area that emits according to the blackbody temperature radiation. The remaining density area of the screen, which is metallic and highly reflective, reflects radiation according to the ambient temperature. Thus, a perforated screen with a controlled hole density allows us to control the emissivity of a commercial blackbody.

The “screen approach” is based on the following assumptions: thermal equilibrium; conservation of energy; Kirchhoff Law; an opaque blackbody; a reflecting plate; and a perforated screen.

The sum of absorptivity, reflectivity, and transmissivity coefficients are equal to 1. Since the blackbody, plate, and the parts of the screen with no holes are opaque, τ = 0.

Emissivity *ε* is a measure of how much thermal radiation a body emits to its environment. For an object in thermal equilibrium with its environment (steady-state conditions, stable temperature, no net heat transfer) the absorptivity is exactly equal to the emissivity (α = ε).

**Figure 1:**
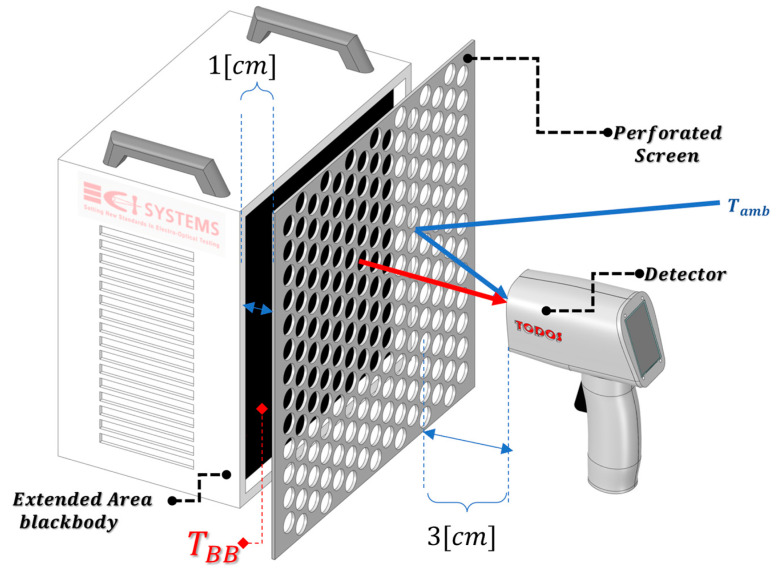
Grey body measurement setup using an extended area blackbody and perforated screen between the blackbody and the detector. *T_BB_* is the blackbody temperature and *T_amb_* is the ambient temperature.

Figure 1 presents the schematic measurement setup to measure an extended area blackbody and perforated screen with a radiometer.

The sensor in Figure 1 is a Digital TMOS with two optical channels, described in Section 4.

The perforated holes must be uniformly distributed, the hole diameter must be small to average, and there must be an adequate number of holes within the field of view of the sensor. At the same time, the hole diameter should be larger than the wavelength of the radiation by a factor of at least 1000. In this study, we focus on IR radiation around 10 µm, and therefore holes around 1 mm in diameter were adequate.

We refer to the above setup (Figure 1) as a grey body and define the effective emissivity of the measured blackbody with a perforated screen as follows:

(4) $$\varepsilon_{eff}=\frac{S_{screen}\left(T_{BB},T_{PTAT},T_{amb}\right)-S_{mirror}\left(T_{amb},T_{PTAT}\right)}{S_{BB}\left(T_{BB},T_{PTAT},T_{amb}\right)-S_{mirror}\left(T_{amb},T_{PTAT}\right)}$$

where $S_{screen}\left(T_{BB},T_{PTAT},T_{amb}\right)$ is the detector signal measured facing the screen between the blackbody, as shown in Figure 1, $S_{BB}\left(T_{BB},T_{PTAT},T_{amb}\right)$ is the detector signal measured facing the blackbody at the same temperature without the screen, and $S_{mirror}\left(T_{amb},T_{PTAT}\right)$ is the signal detector facing a highly reflective plate such as a mirror or metallic with low emissivity. $T_{PTAT}$ is the temperature of the circuit known as proportional-to-absolute-temperature, which measures the temperature of the sensor die.

In Appendix A, it is shown that the signal from the screen is equal to the ratio of the holes of the perforated screen to the total area if the screen and ambient air are at the same temperature. To achieve that, the screen is “soaked” in the ambient temperature (~20 °C), and the measurement is applied rapidly (10 s or fewer).

Thus, the screen with a known percentage of holes can “transform” a near ideal extended area blackbody to a practical grey body with a known emissivity. 

## 3. Manufacturing Perforated Screens with a Range of Emissivity Values

Experimental physicists know that the measured results depend on the small details of the “plumbing”. To illustrate the challenges associated with the fabrication of the perforated screens, let us review how a screen with an emissivity around 0.8 is manufactured.

To obtain an emissivity around 0.8, the holes should fill about 80% of the area. We have been able to achieve such screens only with the photo-etching technology of a commercial vendor [10].

At first, we created the screens mechanically by metal processing, including punching holes. However, the simple act of punching perforated sheets caused distortion, affecting the integrity of the component. For precise calibration, the results were therefore not optimal.

In contrast, manufactured from a single sheet of metal, photochemically etched meshes provide a far more precise, robust, and repeatable solution. As there is no mechanical tooling, the shape and integrity of the metal is maintained to provide a fast, economical, and high-quality result for a wide range of materials. These include typically hard to machine metals such as stainless steel, titanium, and aluminum, which have highly desirable anti-corrosion properties.

With photochemical etching, meshes are manufactured from a single piece of metal, including thicknesses down to 100-micron foil. This gives them greater strength and integrity once they are packaged in a frame as well as a more vertical etching profile. 

We have designed a screen made of 0.1 mm stainless steel with holes with a radius of 1 mm and a pitch between the center of the holes of 2.15 mm. The spec for the product is +/−0.05 mm. The perforated screen provides a ratio of $A_{holes}/A_{plate}\approx 0.8$ between the holes area to the whole plate. To check the reliability of the screen, we ordered two such screens that give almost identical results in the measurements (see Section 6).

**Figure 2:**
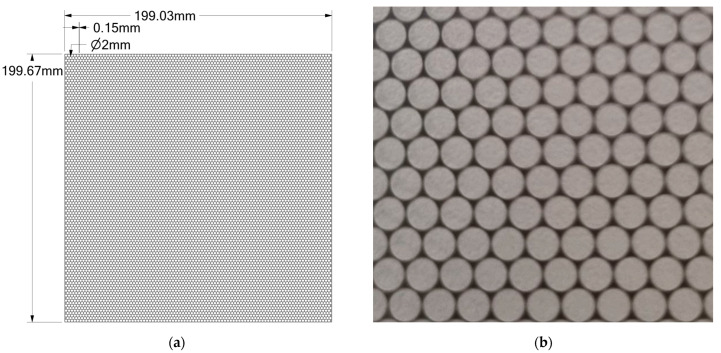
The perforated screen: (**a**) the screen design; (**b**) optical image of part of the processed screen.

Figure 2 illustrates the screen design, with a hole radius of 1 mm and a margin of 0.15 mm.

To preserve the integrity of the thin screen, it is held by a 3D-printed robust frame. It should be noted that the screens may be used for multiple measurements. Hence, the cost of the perforated screens produced by a commercial vendor is not significant.

## 4. The Two-Channel Radiometer Based on the Digital TMOS

### 4.1. Brief Review of the Digital TMOS

The two-channel radiometer based on the Digital TMOS [11] has been reported in Micromachines, 2022, *Toward an Accurate IR Remote Sensing of Body Temperature Radiometer Based on a Novel IR Sensing System Dubbed Digital TMOS* [12]. The TMOS is a micromachined n-MOS CMOS transistor operating at subthreshold and performing as a thermal IR uncooled sensor. The performance metrics and the noise mechanisms of the analog device are reported in [13]. The performance metrics and the noise mechanisms of the digital TMOS are reported in [14]. The measurements results reported in this section and in Section 5 and Section 6 are affected by the overall noise contributions and the computational noise contributed by the modeling.

The unique feature of this device is that it simultaneously employs two sensors with different optical bandpass filters. Accordingly, the measurement yields two power equations with two unknowns: the temperature and the emissivity. By solving these two equations, the effective emissivity is derived.

The device is calibrated with a heuristic model using a high-quality extended area blackbody operating between 30 and 40 °C while the lab temperature is 18–20 °C. 

**Figure 3:**
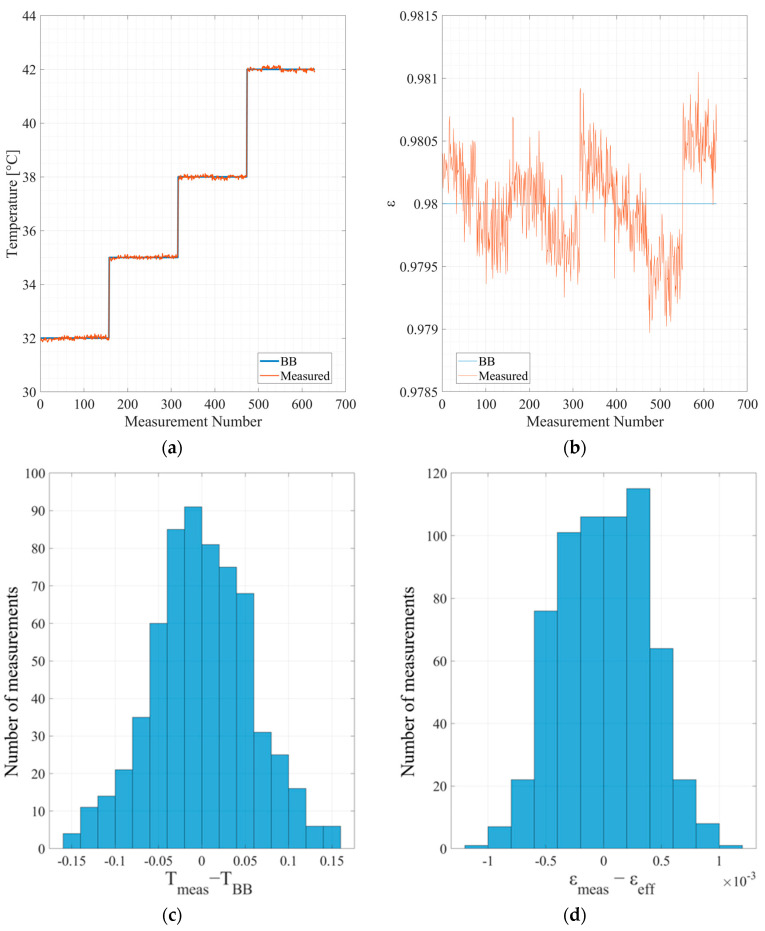
The measured temperature and emissivity of the blackbody: (**a**) blackbody temperature; (**b**) blackbody emissivity; (**c**) histogram of the temperature accuracy in Kelvin; (**d**) histogram of the emissivity accuracy.

Figure 3 exhibits the measured data of the blackbody temperature and emissivity.

### 4.2. The Linearity of the Digital TMOS by Emissivity

**Figure 4:**
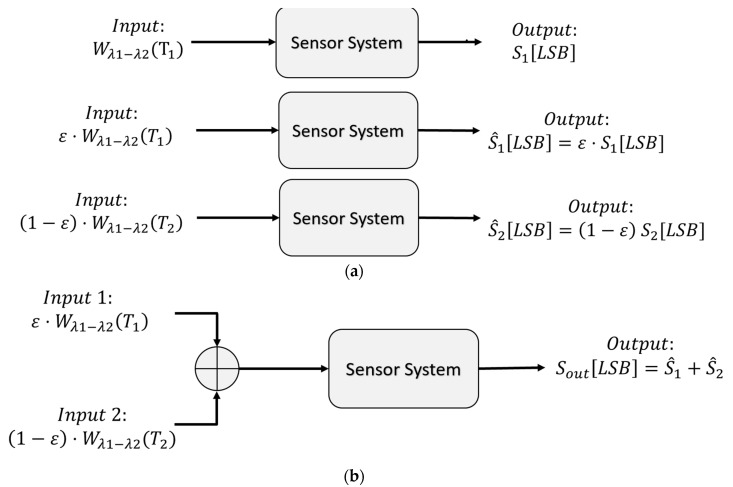
Sensor system emissivity signal linearity: (**a**) single input; (**b**) superposition input.

**Figure 5:**
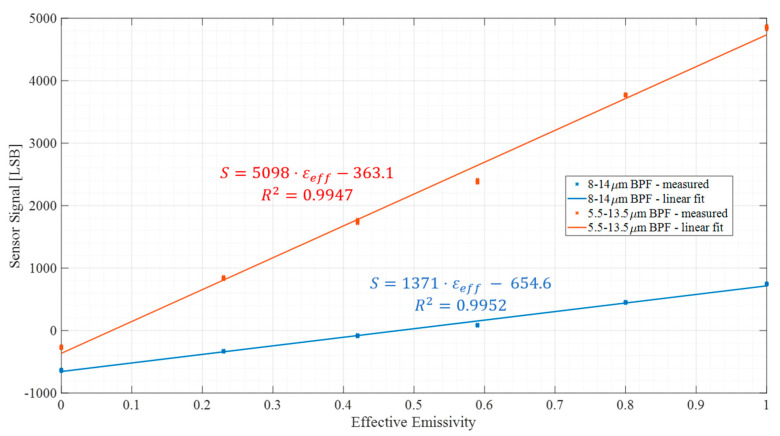
Sensor signal as function of the effective emissivity. The blue line corresponds to the signal measured with an optical band pass filter (BPF) between 8 and 14 µm, and the red line corresponds to the signal measured with an optical BPF between 5.5 and 13.5 µm.

An important feature of the Digital TMOS is the linearity with emissivity. The meaning of linearity is discussed below. Usually, sensors are manufactured with integrated digital readout circuits (ASIC-application-specific integrated circuit). The output signal is in digital units; for example, the sensor in this work measures the signal in the digital units least-significant-bit [LSB]. In other words, if the system input is blackbody radiation with a temperature of $T_{1}$, denoted by $W_{\lambda 1-\lambda 2}\left(T_{1}\right)$ and the output of the sensor system is denoted by $S_{1}\left[LSB\right]$, then if the input is grey body radiation with an emissivity of $\varepsilon_{GB}$ and temperature $T_{1}$, (denoted by $\varepsilon_{GB}W_{\lambda 1-\lambda 2\;}\left(T_{1}\right)$), the output signal is equal to $\varepsilon_{GB}S_{1}\left[LSB\right]$. Similarly, for blackbody radiation with a temperature of $T_{2},$ denoted by $W_{\lambda 1-\lambda 2}\left(T_{2}\right)$, the output signal is denoted by $S_{2}\left[LSB\right].$ Therefore, if the input signal is a superposition of the emitted radiation from a grey body and the reflected radiation from the grey body, the input signal is equal to $\varepsilon_{GB}W_{\lambda 1-\lambda 2\;}\left(T_{1}\right)+\left(1-\varepsilon_{GB}\right)W_{\lambda 1-\lambda 2\;}\left(T_{2}\right)$, where $T_{1}$ is the surface temperature of the grey body and $T_{2}$ is the ambient temperature. Accordingly, the output signal is equal to $\varepsilon_{GB}S_{1}+(1-\varepsilon_{GB})S_{2}$. Figure 4 below illustrates the above explanation:

Figure 5 below shows the linearity of the sensor used in this work as a function of the effective emissivity.

## 5. Measurement of the Effective Emissivity of the Perforated Screen

According to expression (4), three measurements are sufficient to determine the effective emissivity of the perforated screen. The measurement setup is described in Figure 1. The measured signals are obtained by the signal from the blackbody $S_{BB}\left(T_{BB},T_{PTAT},T_{amb}\right)$, the signal of the mirror screen $S_{screen}\left(T_{BB},T_{PTAT},T_{amb}\right)$ and the signal of the mirror $S_{mirror}\left(T_{amb},T_{PTAT}\right)$. The mirror is made of the same sheet of stainless steel from which the screen is made.

**Figure 6:**
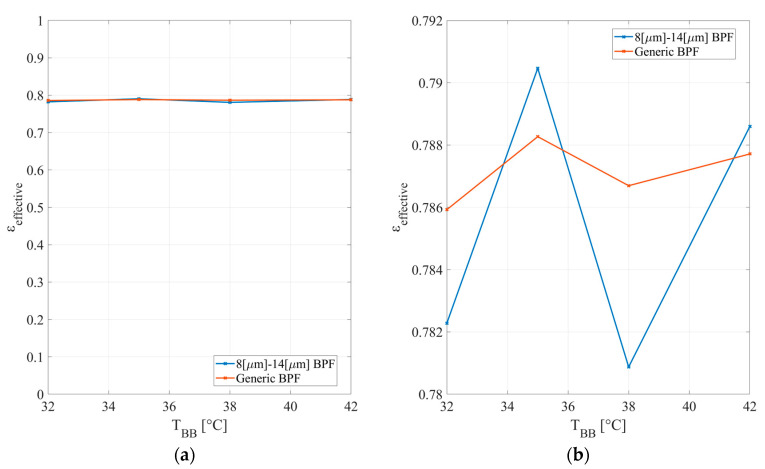
The measured effective emissivity: (**a**) scale between 0 and 1; (**b**) scale with range from 0.78 to 0.792.

The blackbody is operated between 32 and 42 °C. The measurements are performed rapidly in order to control the screen temperature and keep it close to the ambient temperature. Figure 6 shows the measurement results with the Digital TMOS for two optical bandwidths.

It should be noted that for this type of measurement in the lab, a single sensor is adequate to obtain the screen emissivity. The two-channel radiometer is needed just to increase confidence in the results. However, when we actually measure a body with an emissivity of ~0.8 using a radiometer, we cannot perform such three measurements. In that case, we need the two-channel radiometer.

## 6. Validation of the Results Based on the Perforated Screen with Effective Emissivity of 0.786

Validation of the results was based on the grey body, which is the perforated screen, and the blackbody. The measurements reported in Section 6.2 are based on blackbody temperatures, which are not included in the calibration (see Section 6.1).

### 6.1. Calibration

**Figure 7:**
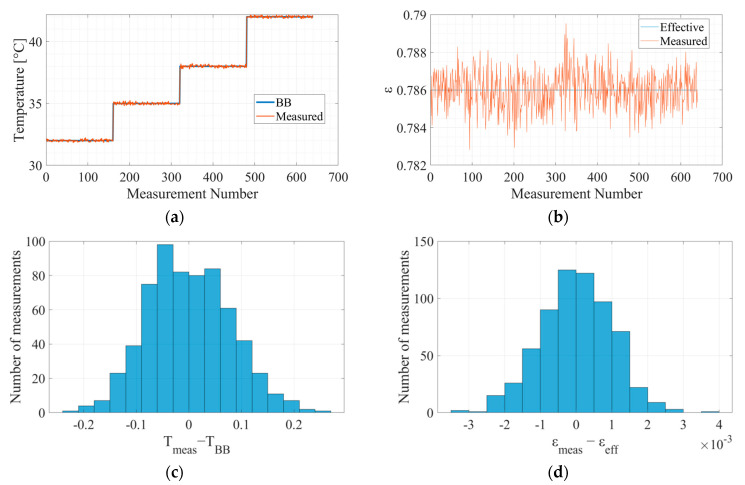
The measured temperature and emissivity of the grey body calibration: (**a**) blackbody temperature; (**b**) effective grey body emissivity; (**c**) histogram of the temperature accuracy in Kelvin; (**d**) histogram of the emissivity accuracy.

For each emissivity range, the model is calibrated and has different model parameters. Figure 7 below exhibits the results of the screen calibration.

### 6.2. Validation

**Figure 8:**
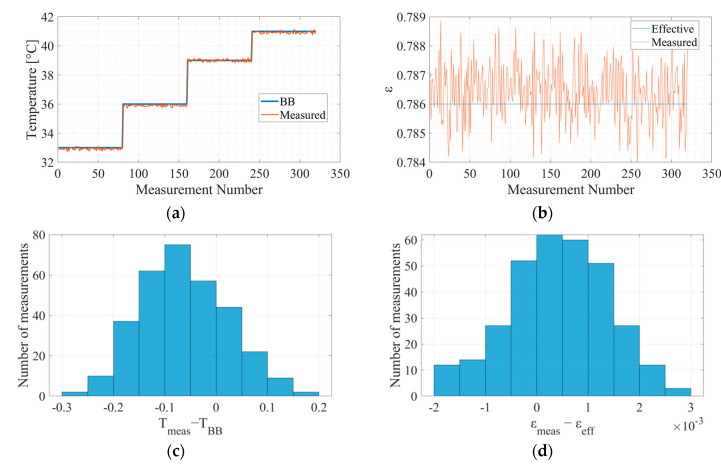
The measured temperature and emissivity of the grey body validation: (**a**) blackbody temperature; (**b**) effective grey body emissivity; (**c**) histogram of the temperature accuracy in Kelvin; (**d**) histogram of the emissivity accuracy.

Figure 8 exhibits the validation results.

The temperature accuracy of the validation set was 0.062 [K], obtained from Figure 8c, and the temperature precision was 0.087 [K] for a single sampled measurement without any averaging to reduce noise. The emissivity accuracy of the validation set was 0.0004244, obtained from Figure 8d, and the emissivity precision was 0.000986 for a single sampled measurement without any averaging to reduce noise.

## 7. Conclusions

The importance of remote sensing of temperature by measuring IR radiation has been recognized in a wide range of industrial, medical, and environmental uses. The advantages of thermography by remote sensing are self-evident. During industrial processing and manufacturing, the measured object may be very hot as well as hazardous. The recent COVID-19 pandemic has increased the need and motivation for accurate and low-cost thermometers that detect human body temperature with high accuracy of 0.1 °C.

This study reports a simple yet very important method for calibrating radiometric measurements using a grey body with a known emissivity as well as temperature. The method and the measurements are obtained with novel methodology as well as the innovative micromachined uncooled infrared sensor, dubbed Digital-TMOS. The Digital TMOS is a low-cost commercial product, it requires low power, and it has a small form factor. In contrast, multispectral radiometers to measure emissivity are offered by CI systems and other commercial companies, but such equipment is bulky and expensive. 

The concept of emissivity has been with the scientific and engineering world since Planck formulated his blackbody radiation law more than a century ago. Nevertheless, emissivity is an elusive concept even for experts. It is a vague and fuzzy concept for the wider community of engineers. 

However, a deeper understanding of the physics of remote IR temperature sensing teaches us that the measured temperature accuracy cannot be achieved without determining the surface emissivity as well. The reported tables of material emissivity are not useful since emissivity may change considerably due to oxidation and aging.

This study describes how to achieve a calibrated and stable emissivity with a blackbody, a perforated screen, and a reliable and linear IR thermal sensor. The paper describes in detail how to obtain the perforated screen as well as how to perform the calibration.

## Figures and Tables

**Figure A1:**
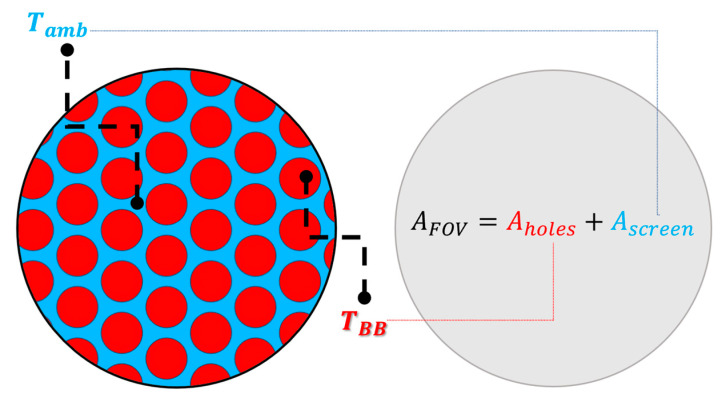
Illustration of the sensor field of view facing the screen in front of the blackbody. The red color illustrates the holes area in the sensor filed of view, which relates to radiance with temperature of the black body surface, *T_BB_*. The blue color illustrates the screen metal area in the sensor filed of view, which relates to radiance with ambient temperature *T_amb_*.

## Data Availability

The data that support the findings of this study are available from the corresponding author, Y.N, upon reasonable request.

## Appendix A. Effective Screen Emissivity

The measured signal with the perforated screen is denoted by *S_screen_*, and the screen signal is a function of the blackbody temperature *T_BB_*, the screen temperature *T_screen_*, the PTAT temperature *T_PTAT_*, and the ambient temperature-*T_amb_*. The measured signal is proportional to the incident radiation and the emitted radiation multiplied by the read-out circuitry gain (denoted by G). Therefore, the signal can be written as:

(A1) $$S_{screen}\left(T_{BB},T_{\mathrm{screen}},T_{PTAT},T_{amb}\right)\;=G\left[\underset{emitted-directly-through-holes}{\underbrace{\frac{A_{holes}}{A_{FOV}}\cdot W\left(T_{BB}\right)}}+\underset{emitted-directly-by-screen}{\underbrace{\varepsilon_{screen}\frac{A_{screen}}{A_{FOV}}\cdot W\left(T_{screen}\right)}}+\underset{reflected-by-screen}{\underbrace{\left(1-\varepsilon_{screen}\right)\frac{A_{screen}}{A_{FOV}}\cdot W\left(T_{amb}\right)}}-W\left(T_{PTAT}\right)\right]$$

where *A_FOV_* is the total field of view area of the sensor. *A_holes_* is the holes area in the sensor field of view, and *A_screen_* is the plate area without the holes in the sensor field of view. Figure A1 illustrates those areas.

For an ideal blackbody, the signal from the blackbody, which is denoted by *S_BB_*, is equal to:

(A2) $$S_{BB}\left(T_{obj},T_{PTAT}\right)=G\times \left[W\left(T_{obj}\right)-W\left(T_{PTAT}\right)\right]$$

Similarly, the mirror (ambient) signal is equal to:

(A3) $$S_{amb}\left(T_{amb},T_{PTAT}\right)=G\times \left[W\left(T_{amb}\right)-W\left(T_{PTAT}\right)\right]$$

Therefore, the numerator of Equation (4) is equal to:

(A4) $$S_{screen}\left(T_{obj},T_{PTAT}\right)-S_{amb}\left(T_{amb},T_{PTAT}\right)=\;G\times \left[\underset{emitted-directly-through-holes}{\underbrace{\frac{A_{holes}}{A_{FOV}}W\left(T_{BB}\right)}}+\underset{emitted-directly-by-screen}{\underbrace{\varepsilon_{screen}\frac{A_{screen}}{A_{FOV}}W\left(T_{screen}\right)}}+\underset{reflected-by-screen}{\underbrace{\left(1-\varepsilon_{screen}\right)\frac{A_{screen}}{A_{FOV}}W\left(T_{amb}\right)}}-W\left(T_{PTAT}\right)\right]-G\times \left[W\left(T_{amb}\right)-W\left(T_{PTAT}\right)\right]\;=G\times \left(\frac{A_{holes}}{A_{FOV}}W\left(T_{BB}\right)+\varepsilon_{screen}\left(1-\frac{A_{holes}}{A_{FOV}}\right)W\left(T_{screen}\right)+\left(1-\frac{A_{holes}}{A_{FOV}}\right)W\left(T_{amb}\right)-\varepsilon_{screen}\left(1-\frac{A_{holes}}{A_{FOV}}\right)W\left(T_{amb}\right)-W\left(T_{amb}\right)\right)=G\times \left(\frac{A_{holes}}{A_{FOV}}W\left(T_{BB}\right)+\varepsilon_{screen}\left(1-\frac{A_{holes}}{A_{FOV}}\right)W\left(T_{screen}\right)-\varepsilon_{screen}\left(1-\frac{A_{holes}}{A_{FOV}}\right)W\left(T_{amb}\right)-\frac{A_{holes}}{A_{FOV}}W\left(T_{amb}\right)\right)=G\times \left(\frac{A_{holes}}{A_{FOV}}\left(W\left(T_{BB}\right)-W\left(T_{amb}\right)\right)+\varepsilon_{screen}\left(1-\frac{A_{holes}}{A_{FOV}}\right)\left(W\left(T_{screen}\right)-W\left(T_{amb}\right)\right)\right)$$

Therefore, the enumerator of Equation (4) is equal to:

(A5) $$S_{BB}\left(T_{obj},T_{PTAT}\right)-S_{amb}\left(T_{amb},T_{PTAT}\right)=G\times \left[W\left(T_{BB}\right)-W\left(T_{amb}\right)\right]$$

The effective emissivity can be derived by dividing Equations (A4) to (A5):

(A6) $$\varepsilon_{eff}=\frac{G\times \left[\frac{A_{holes}}{A_{FOV}}\left(W\left(T_{BB}\right)-W\left(T_{amb}\right)\right)+\varepsilon_{screen}\left(1-\frac{A_{holes}}{A_{FOV}}\right)\left(W\left(T_{screen}\right)-W\left(T_{amb}\right)\right)\right]}{G\times \left[W\left(T_{BB}\right)-W\left(T_{amb}\right)\right]}=\frac{A_{holes}}{A_{FOV}}+\varepsilon_{screen}\left(1-\frac{A_{holes}}{A_{FOV}}\right)\frac{W\left(T_{screen}\right)-W\left(T_{amb}\right)}{W\left(T_{BB}\right)-W\left(T_{amb}\right)}$$

Table A1 summarizes the effect of the screen temperature upon the effective emissivity.

**Table A1:** Effective emissivity with the screen approach.

$T_{screen}$	$\varepsilon_{eff}$
$T_{screen}=T_{amb}$	$\frac{A_{holes}}{A_{FOV}}$
$T_{screen}\neq T_{amb}$	$\frac{A_{holes}}{A_{FOV}}+\varepsilon_{screen}\left(1-\frac{A_{holes}}{A_{FOV}}\right)\frac{W\left(T_{screen}\right)-W\left(T_{amb}\right)}{W\left(T_{BB}\right)-W\left(T_{amb}\right)}$

## Appendix B. Other Methods for Emissivity Measurement and Compensation

For example, we refer to four approaches to measure emissivity:

(1) Reflective cavity pyrometers:
  One method is placing a mirror around the aperture of the radiometer. This mirror reflects radiation from the target surface back to the target, effectively replacing some of the ambient radiation with radiation from the target itself. If the mirror were ideal, r = 1 and infinitely large, and if the aperture of the radiometer were infinitesimally small, this would give an effective emissivity of 1 for the target surface. For less reflective and smaller mirrors, the emissivity is only increased somewhat closer to 1. See, for example, [15].

(2) Reflective cavity pyrometers with two probes:
  A variation of method (1) is using two different mirrors, which allows two measurements at two different emissivity enhancement levels. The two measurements can be extrapolated to the signal levels, which would correspond to an emissivity enhancement of 1. See, for example, [16].

(3) “Self-reflectance’’ method of emissivity correction using a chopper:
  This method is similar to method (2), except the mirror is replaced by a chopper inside the optical system. This requires a complicated optical system and is used mainly for group-III nitride MOCVD temperature measurements [17].

(4) Active pyrometry with emissivity compensation:
  Electro-optical testing companies sell a temperature probe similar to method (3), but instead of using a chopper to reflect radiation back to the source, they radiate a separate chopped radiation source at the target and also measure the target’s self-emission when the internal light source is off. For example, see [18].
